# Fine mapping of *CscpFtsY*, a gene conferring the yellow leaf phenotype in cucumber (*Cucumis sativus* L.)

**DOI:** 10.1186/s12870-022-03922-0

**Published:** 2022-12-06

**Authors:** Gaohui Zha, Juan Yin, Feng Cheng, Mengfei Song, Mengru Zhang, Hesbon Ochieng Obel, Yi Wang, Jinfeng Chen, Qunfeng Lou

**Affiliations:** State Key Laboratory of Crop Genetics and Germplasm Enhancement, College of Horticulture, Nanjing Agricultural University, Weigang Street No.1, Nanjing, 210095 China

**Keywords:** cpSRP, cpFtsY, Gene cloning, Photosynthesis, Leaf color mutant, Cucumber

## Abstract

**Background:**

Leaf color mutants are ideal materials to study pigment metabolism and photosynthesis. Leaf color variations are mainly affected by chlorophylls (Chls) and carotenoid contents and chloroplast development in higher plants. However, the regulation of chlorophyll metabolism remains poorly understood in many plant species. The chloroplast signal-recognition particle system is responsible for the insertion of the light-harvesting chlorophyll a/b proteins (LHCPs) to thylakoid membranes, which controls the chloroplast development as well as the regulation of Chls biosynthesis post-translationally in higher plants.

**Results:**

In this study, the yellow leaf cucumber mutant, named *yl*, was found in an EMS-induced mutant library, which exhibited a significantly reduced chlorophyll content, abnormal chloroplast ultrastructure and decreased photosynthetic capacity. Genetic analysis demonstrated that the phenotype of *yl* was controlled by a recessive nuclear gene. Using BSA-seq technology combined with the map-based cloning method, we narrowed the locus to a 100 kb interval in chromosome 3. Linkage analysis and allelism test validated the candidate SNP residing in CsaV3_3G009150 encoding one homolog of chloroplast signal-recognition particle (cpSRP) receptor in Arabidopsis, cpFtsY, could be responsible for the yellow leaf phenotype of *yl*. The relative expression of *CscpFtsY* was significantly down-regulated in different organs except for the stem, of *yl* compared with that in the wild type (WT). Subcellular localization result showed that CscpFtsY located in the chloroplasts of mesophyll cells.

**Conclusions:**

The *yl* mutant displayed Chls-deficient, impaired chloroplast ultrastructure with intermittent grana stacks and significantly decreased photosynthetic capacity. The isolation of CscpFtsY in cucumber could accelerate the progress on chloroplast development by cpSRP-dependant LHCP delivery system and regulation of Chls biosynthesis in a post-translational way.

**Supplementary Information:**

The online version contains supplementary material available at 10.1186/s12870-022-03922-0.

## Background

As a key visible feature of leaves, leaf color is a reliable marker for plant breeding [1]. Due to variations in the Chls and carotenoid contents in the chloroplast, plant leaves can exhibit a wide spectrum of hues. In higher plants, chloroplasts are essential organelles for photosynthesis. Chloroplast formation is influenced by environmental factors such as light and temperature and the coordinated expression of nuclear and plastid genes [2]. Chls metabolism is a highly coordinated process involving a series of cooperative reactions catalyzed by a variety of enzymes [3, 4]. Recent research has helped to better define the Chls metabolism pathway, specifically by identifying the genes involved.

Chloroplasts are the major sites of Chls and carotenoid biosynthesis and degradation, which accounts for the variation in leaf color in plants. The biogenesis and development of chloroplasts require coordinated protein assembly and signaling networks between the chloroplast and nucleus [5]. The abnormal structure of chloroplast can be one major reason for leaf color variation. It has been reported that the variations in leaf color can be caused by defects in RNA-editing factor [6, 7], plastid ribosomal proteins [8], and chromatin remodeling factor [9] which are related to chloroplast biogenesis or development.

Leaf color mutants are valuable genetic resources for deciphering the molecular mechanisms underlying chloroplast development and Chls metabolic mechanism in plants [10]. The Chls metabolic process in Arabidopsis is exceptionally well understood, with 27 genes cloned and well-defined for all 15 steps and 15 types of enzymes involved [3, 11]. Any alteration of the genes encoding enzymes in the chlorophyll metabolism could cause leaf color mutation through changes in Chls metabolism [3, 12–14]. The Chls metabolism pathway can be divided into three processes: the biosynthesis of Chl a from glutamate, the interconversion of Chl a and Chl b, and the degradation of Chls [3]. A number of genes involved in chlorophyll metabolism pathway have been identified and cloned using leaf color mutants in Arabidopsis [10], rice [15], and maize [16].

Chls content are regulated not only by modulating the amount of Chls metabolism-related enzymes in a transcriptional and translational manner but also required by post-translational regulators to promote Chls homeostasis by adjusting the balance between Chls biosynthesis and degradation during leaf development [17]. One of the chloroplast signal-recognition particles (cpSRP) pathway members, cpSRP43 had been demonstrated that it regulates Chls metabolism pathway as a post-translational role by directly binding and preventing aggregation of glutamyl-tRNA reductase (GluTR), a rate-limiting enzyme in Chls biosynthesis [18]. In chloroplast, cpSRP system is responsible for the transport of nucleus-encoded light-harvesting Chl a/b binding proteins (LHCPs) in the stroma of the chloroplast and their assembly to the thylakoid membranes [18, 19]. Chls together with carotenoid noncovalently binds LHCPs to form a stable complex, light-harvesting complexes (LHCs) where photoreaction occurs. Whereas the free Chls that do not bind LHCPs to have a marked tendency to generate reactive oxygen species (ROS) in the light,causes oxidative damage to proteins, DNA, and lipids [18, 20]. Then the LHCPs bind Chls and carotenoids to form a stable light-harvesting complex (LHCs) where photo-action occurs [21]. It has been demonstrated that cpSRP43 effectively protects several Chls biosynthesis-related proteins from heat-induced aggregation and enhances their stability during leaf greening and heat shock [22]. Therefore, the spatiotemporal coordination between the cpSRP machinery and Chls biosynthesis is an important issue to be illuminated in the future.

Cucumber (*Cucumis sativus* L.) is a popular and one of the most important vegetable crops in the world. Although a number of studies on Chls metabolism pathway, chloroplast development and other factors that affect leaf color had been performed in cucumber (documented in Table S1). However, the lacking reports of defects on cpSRP machinery that cause leaf color mutants hinders the understanding of Chls regulation network on the post-translational pathway. Here the yellow-leaf cucumber (*yl*) we identified in the EMS-induced mutant library, exhibits a yellow leaf phenotype with a decrease in Chls and carotenoid content and abnormal chloroplast structure and the photosynthesis capability of *yl* is significantly lower than WT. BSA-seq and map-based cloning narrow the *yl* locus within a 100 Kb physical interval in chromosome 3. Only one available SNP (G- > A) was identified in the candidate region located in the second exon of CsaV3_3G009150 encoding one homolog of chloroplast signal recognition particle receptor in *Arabidopsis thaliana*, cpFtsY. So the candidate gene was named Cscpftsy. This study could help to unravel the Chls metabolism in a post-translational manner and provide valuable material to study cpSRP member’s role in horticultural crops.

## Results

### Phenotypic characterization of *yl* mutant

**Fig. 1:**
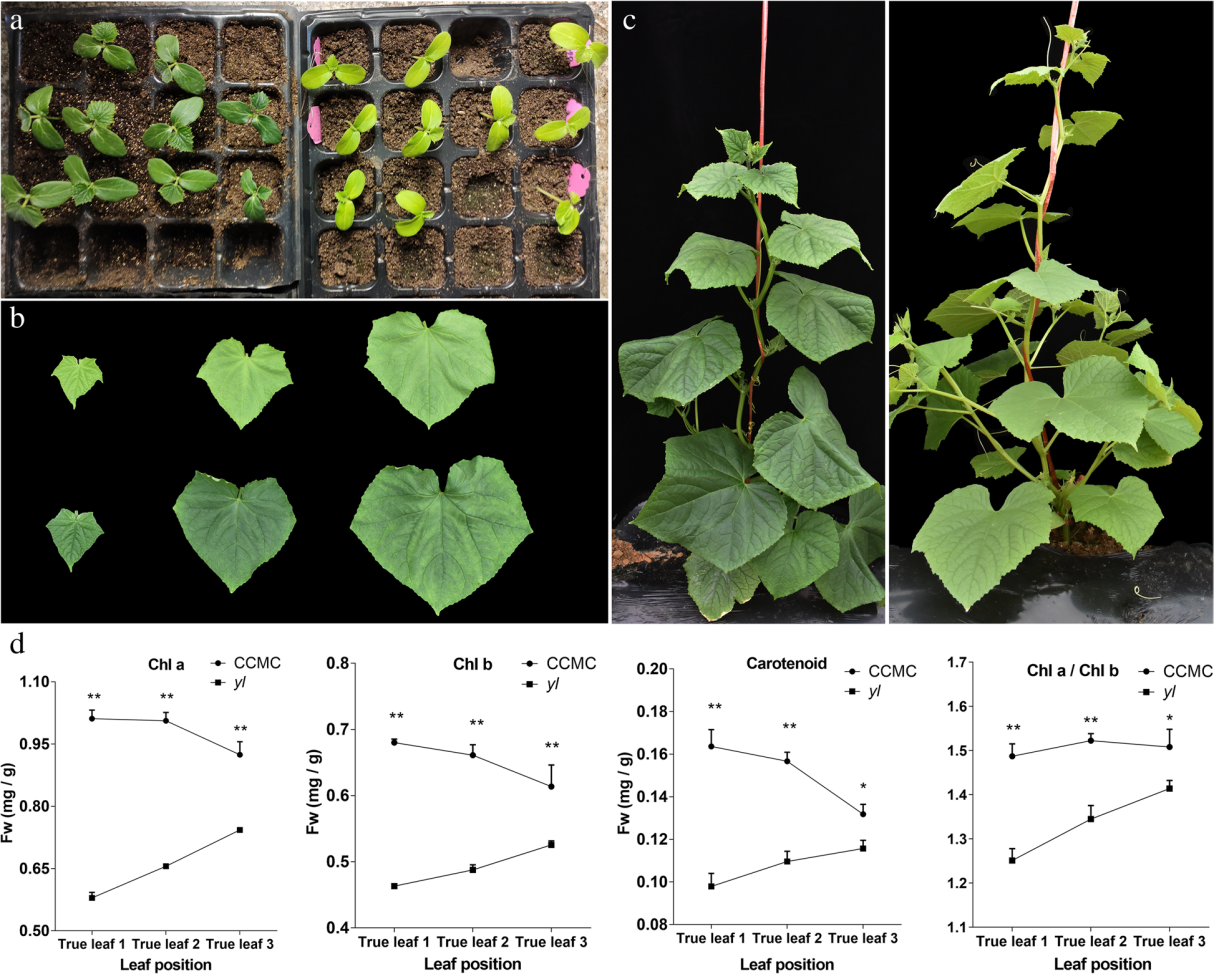
Plant morphology, leaf color and pigment content of of CCMC and *yl*. **a** Phenotype of CCMC (left) and *yl* (right) in at the cotyledon stage. Scale bar, 5.5 cm. **b** Color of the top three leaves collected from *yl* (up) and CCMC (down) plants shown in c. Scale bar, 4 cm. **c** The whole plant phenotype of CCMC (left, scale bar, 15 cm.) and *yl* (right, scale bar, 12 cm.) at the vegetative stage. **d** Pigments contents in fresh leaves between CCMC and *yl* in the top three position leaves. Error bars indicate the standard error. * and ** *P* < 0.05 and *P* < 0.01, respectively (Multiple *t*-test)

A yellow leaf (*yl*) mutant was discovered from the M_2_ families derived from an EMS-mutagenized cucumber CCMC population. Compared with CCMC, the *yl* displayed a light yellow leaf, especially in cotyledon (Fig. 1a, c). The characteristic of the gradual yellow leaves of *yl* are that the young leaves are more yellow than the old ones (Fig. 1b). The *yl* showed relatively weaker and slower growth but stronger branching than CCMC (Fig. 1c). During the reproductive phase, the heights of *yl* and CCMC are about 159.7 ± 1.38 cm and 110.0 ± 5.33 cm, respectively. However, *yl* developed more but slim veins than CCMC. At maturity, *yl* mutant blossomed, bear fruits and harvest seeds normally under field conditions.

Chls content is one of the major factors directly affecting leaf color in plants, therefore the pigment contents between CCMC and *yl* in the top three leaves were measured. The result showed that the contents of Chl a, Chl b, total carotenoid and Chl a/b ratio in *yl* are respectively significantly reduced in the top three leaves compared with CCMC (Fig. 1d).

**Fig. 2:**
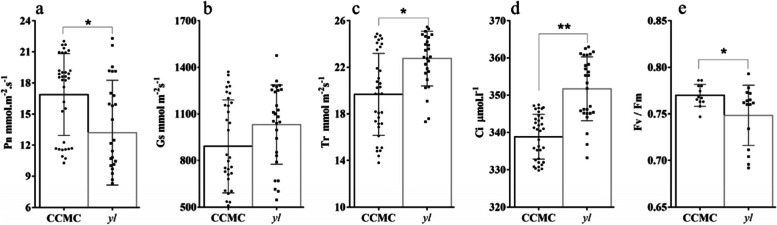
Photosynthesis parameters between CCMC and *yl* in the top three position leaves. **a** Net CO2 assimilation rate (Pn). **B** Stomatal conductance (Gs). **C** Transpiration rate (Tr). **d** Intercellular CO2 concentration. **e** Maximal photochemical efficiency of PSIIin the dark (Fv / Fm). Dots indicate the measurement values of marked individuals. Error bars indicate the standard error. * and ** *P* < 0.05 and *P* < 0.01, respectively (Student’s *t—*test)

Several key photosynthetic parameters were compared between *yl* and CCMC plants. Except for the stomatal conductance (Gs), there are significant differences in net CO_2_ assimilation rate (Pn), transpiration rate (Tr), intercellular CO_2_ concentration (Ci) and Fv / Fm between *yl* and CCMC. The *yl* showed a significantly lower net CO_2_ assimilation rate than CCMC, while the stomatal conductance (Gsw), Tr and Ci were all significantly higher in *yl* compared with CCMC (Fig. 2).

### The chloroplast ultrastructure observation by TEM

**Fig. 3:**
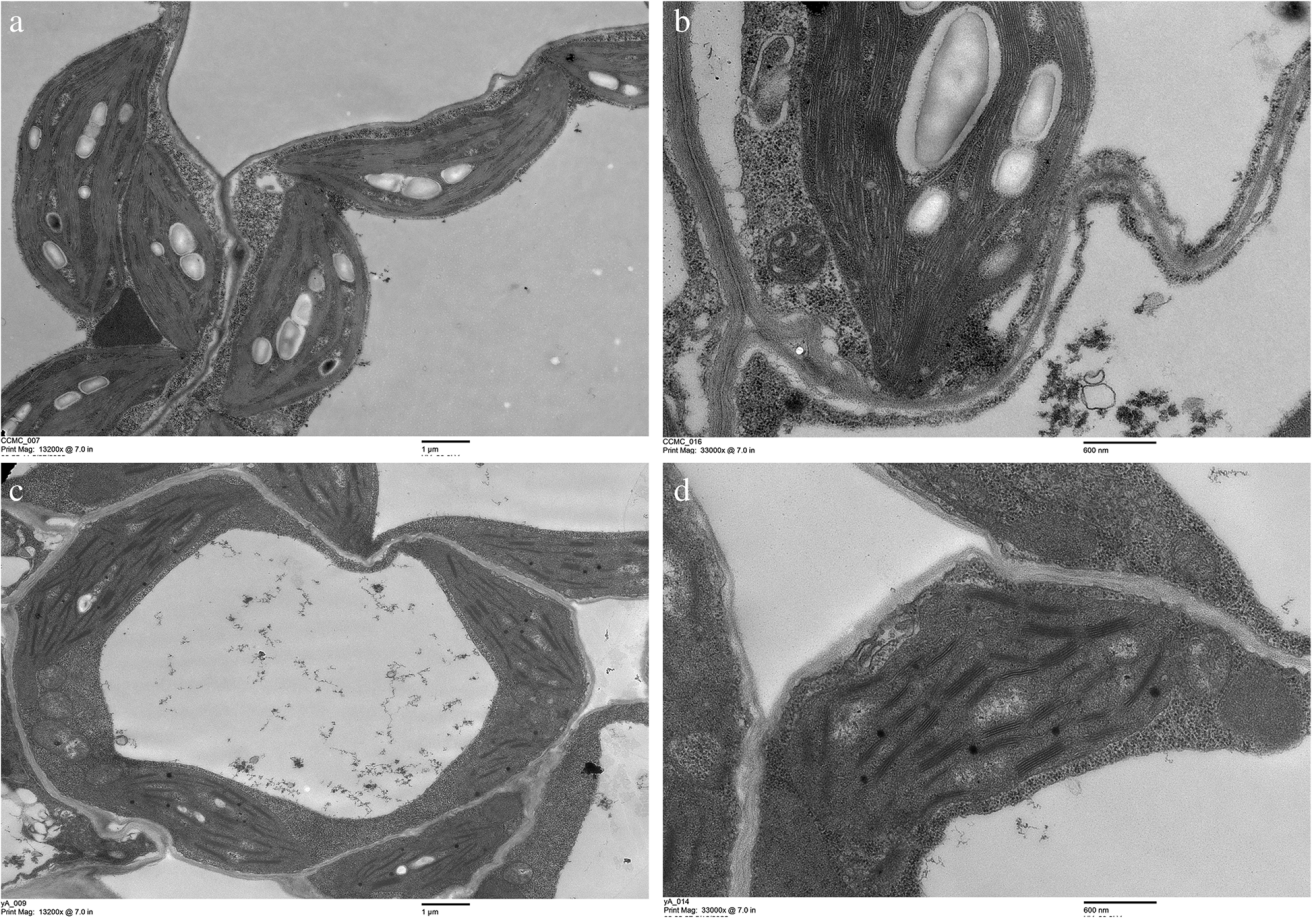
Chloroplast ultrastructure between CCMC and *yl*. **a**,**c** Mesophyll cell ultrastructure of CCMC and *yl*. **b**,**d** Chloroplast ultrastructure of CCMC and *yl*. cp, chloroplast; sg, starch grain; g, grana; pg, plastoglobulus

To determine whether there are defects in the chloroplast in *yl*, the ultrastructure of *yl* and CCMC chloroplasts were examined using transmission electron microscopy (TEM). It can be found that the single chloroplast of CCMC is oval (Fig. 3a), while the chloroplast of *yl* is irregularly shaped (Fig. 3c). The lamellar structure of the chloroplast of CCMC is dense and clear (Fig. 3b), and the lamellar structure of chloroplast of *yl* is discontinuous and stacked unevenly (Fig. 3d). There are more and larger starch grains in CCMC than that in *yl* (Fig. 3a, c). The chloroplasts of *yl* contained a significant number of plastoglobules that were not present in the structure of the CCMC chloroplasts (Fig. 3d).

### Identification of the candidate gene *Cscpftsy*

To study the inheritance pattern of *yl* phenotype, we constructed two F_2_ populations from the crosses between the *yl* with CCMC and Hazerd. All the F_1_ plants displayed wild-type phenotypes. Among the 150 CCMC × *yl* F_2_ plants, there were 116 WT and 34 MT which is consistent with the 3:1 Mendelian inheritance law (Table 1). Among the 982 Hazerd × *yl* F_2_ population, 723 had WT phenotype, whereas 259 exhibited the MT phenotype (Table 1). Therefore, the yellow leaf phenotype in the *yl* was controlled by a recessive nuclear gene.

**Table 1:** Segregation analysis of leaf color phenotype

Population	Green leaf	Yellow leaf	Total number of plants	Expected ratio	χ^2^	p value
CCMC × *yl*	116	34	150	3: 1	0.44	0.51
Hazerd × *yl*	723	259	982	3: 1	1.31	0.25
χ^2^ (0.05, 1) = 3.84						

BSA-seq results showed that there are 11.7 billion raw reads from the WT pool and 10.4 billion raw reads from the mutant pool. The clean reads coverage were about 94.0% and 95% respectively in WT and MT pools. And the error rate were both 0.03% in WT and MT pools. BSA-seq Delta SNP-index Manhattan picture showed that there is a peak at chromosome 3 between 1 and 8 Mb that exceeds the 95% threshold line (Fig. S1). To eliminate background noise, we calculated the ΔSNP index. Considering the preference of EMS-induced mutagenesis, we selected a subset of SNPs with the following criteria for further research: (1) base mutation with transitions of G–A or C–T; (2) SNP-index of MT pool = 1; (3) the SNP resides in exons or splicing site. This screening resulted in only one SNP which was located in the second exon of the CsaV3_3G009150 gene, and it was considered as the candidate SNP which is responsible for the *yl* phenotype.

**Fig. 4:**
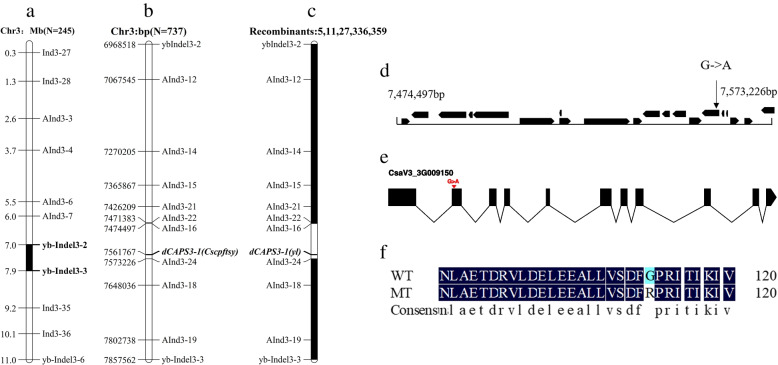
Identification of *Cscpftsy* for yellow leaf phenotype. **a** Initial mapping with 245 F_2_ individuals placed *Cscpftsy* at 0.9 Mb physical interval flanked by yb-Indel3-2 and yb-Indel3-3 in chromosome 3. **b** Further mapping with 737 F_2_ individuals narrowed *Cscpftsy* down a 100 Kb physical interval flanked by AInd3-16 and AInd3-24, **c** dCAPS3-1 was developed based the candidate SNP to identify the co-segregation among five recombinants. **d** 20 candidate genes in the fine mapping region. **e** The structure of candidate gene CsaV3_009150 and candidate SNP(G-A), black boxes mean exons and lines mean introns. **f** The candidate SNP changed Glycine (Gly) to Arginine (Arg)

To map the *yl* locus, an F_2_ mapping population including 245 individuals was generated from a cross between *yl* mutant and Hazerd. The *yl* locus was mapped to an interval around 900 kb between markers yb-Indel3-2 and yb-Indel3-3 on chromosome 3 (Fig. 4a). In order to narrow down the candidate region, 9 polymorphic InDel markers were obtained between the yb-Indel3-2 and yb-Indel3-3. These 11 markers were used to genotype individuals in F_2_ population with a total population of 737 (Fig. 4b). After the genotypes of the recombinant individuals were identified (Fig. 4c), the *yl* locus was delimited to a 100 kb genomic region, which contains 20 genes within AInd3-16 and AInd3-24 (Fig. 4d).

There are 20 genes in the fine mapping region (Table S3), however, only one SNP residing the CsaV3_3G009150 was found (Table S4). Therefore, we developed the dCAPS3-1 to examine the linkage with their phenotype among the mutant individuals (recombinants are contained). As expected, the genotype is exactly consistent with their phenotype (Fig. S2). We also used dCAPS3-1 to perform an allelism test in eighty cucumber natural populations (Fig. S2). And the result showed that all these lines carried the same allele as the wild-type CCMC and Hazerd at this locus, again confirming this SNP within the CsaV3_3G009150 as the causal mutation for the yellow leaf in *yl*.

Based on the Cucurbit database we found that the candidate gene CsaV3_3G009150 with 11 exons and 10 introns (Fig. 4e), encodes a chloroplast signal-recognition particle receptor cpFtsY, one homolog of cpFtsY in Arabidopsis, so the gene was named *CscpFtsY*. The candidate SNP (G- > A) is located at the second exon and changed the 112th Gly to Arg of CscpFtsY (Fig. 4f).

### Expression and homology analysis of CscpFtsY

**Fig. 5:**
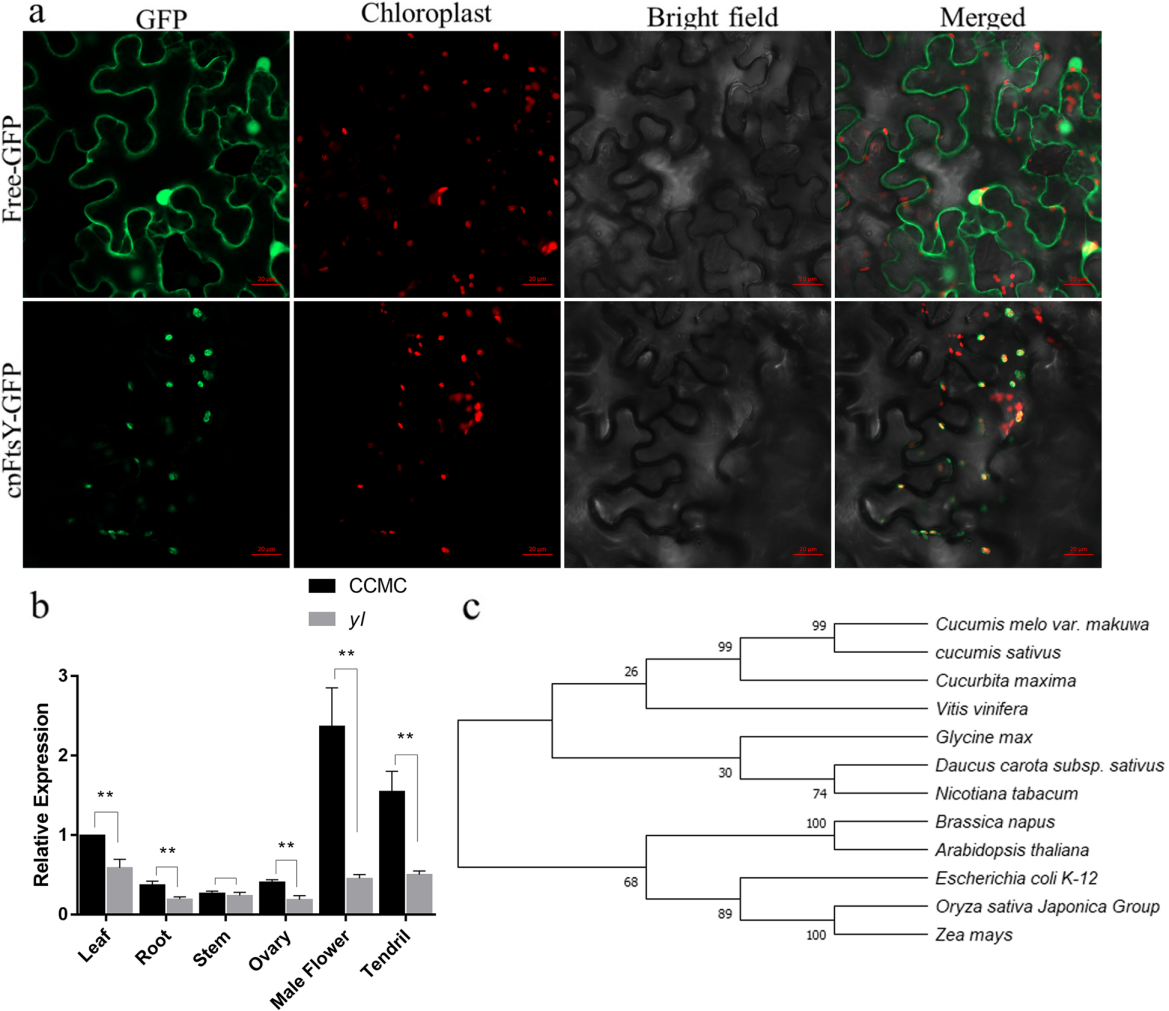
Expression pattern and homology analysis of *CscpFtsY*. **a** *CscpFtsY-GFP* fusion vector was transiently expressed in tobacco cell, and GFP signal was observed by confocal fluorescence microscopy. **b** Relative expression of *CscpFtsY* in different tissues of CCMC and *yl* plants measured by qRT-PCR. Each experiment with three replications, error bars indicate the standard error. ** *P* < 0.01 (Multiple *t* test). **c** Phylogenetic analysis of CscpFtsY in cucumber and its homologs in other species. The phylogenetic tree was constructed by the neighbor-joining method built in MEGA X, and the inferred phylogeny was tested by bootstrap analysis with 1000 replicate datasets. Numbers at the tree forks indicated bootstrap values

To explore the location site of CscpFtsY in cells, subcellular localization was conducted with tobacco leaves. The fluorescence results of infected tobacco leaves displayed that the CscpFtsY-GFP fusion proteins were located in the chloroplasts, while free-GFP was observed in the nucleus and cell membrane (Fig. 5a).

In order to quantify the expression level of *CscpFtsY* in different organs in *yl* and CCMC, we conducted qRT-PCR assays in different organs of *yl* and CCMC. Except for the stem, the relative expression level of *CscpFtsY* in different organs was all lower in *yl* compared with CCMC (Fig. 5b).

To better understand the phylogenetic relationships of cpFtsY protein, a phylogenic tree was generated using the amino sequences from CscpFtsY and 11 other homologous proteins among other species (Fig. 5c). The sequence similarity between the cucumber CscpFtsY and its homologs suggested these cpFtsY genes may serve conserved domains in these organisms (Fig. S3). And there were yellowish leaf color mutants found in Arabidopsis and Maize defected in the homologous genes of *CscpFtsY* [23, 24].

## Methods

### Plant materials

The *yl* mutant was identified from an M_2_ family derived from an EMS-mutagenized cucumber inbred line CCMC (Changchunmici), a common inbred line in North China, which exhibits dark green leaves. Two segregating populations from the crosses of *yl* (male) with the wild-type line CCMC and an inbred line Hazerd (European greenhouse type, which exhibits green leaves and strong branching ability) were developed for genetic analysis, gene mapping and cloning. All plants were planted in the greenhouse at Bai Ma farm experimental field station of Nanjing Agricultural University, Nanjing, China.

The environmental temperature ranged from 25 to 35 degree Celsius, and the light intensity was about 65,000 Lx during the plant growth and phenotypic identification in the greenhouse. The morphological characteristics such as the leaf color of *yl* and CCMC could be visually recognized during the whole development stage.

### Phenotype characterization, pigment extraction and photosynthetic parameter measurement

Phenotypes of the *yl* and CCMC were recorded and leaf color was an identifiable trait distinguishing wild type (WT) from mutant type (MT). The phenotype was identified visually during the whole developmental stages. We conducted phenotyping at three levels, that is, one time the cotyledon stage and two times during the vegetative growth stage. The number of yellow-leaf individuals and green-leaf individuals in these two populations (CCMC × *yl* F_2_ and Hazerd × *yl* F_2_) were counted.

The samples for Chls and carotenoid extraction were collected at the vegetative growth stage (Fig. 1c). Each sample (0.2 g fresh leaves) was collected at the 1st, 2nd and 3rd true leaves position, respectively. Then each sample was quickly cut into small pieces and immersed in 40 ml ethanol with 5% water (v/v) for 12 h. As the tissue became white, the absorbance value of the extraction solution was measured by a microplate reader. The computation of Chls and carotenoid contents were performed following the formula derived from Lichtenthaler HK et al. [25] as follows:

$${\mathrm{C}}_{\mathrm{a}}\left(\frac{\mathrm{\mu g}}{\mathrm{ml}}\right)=13.36\times {\mathrm{OD}}_{664.1}-5.19\times {\mathrm{OD}}_{648.6}$$

$${\mathrm{C}}_{\mathrm{b}}\left(\mathrm{\mu g}/\mathrm{ml}\right)=27.43\times {\mathrm{OD}}_{648.6}-8.12\times {\mathrm{OD}}_{664.1}$$

$$\mathrm{Carotenoid }\left(\mathrm{\mu g}/\mathrm{ml}\right)=(1000\times {\mathrm{OD}}_{470}-2.13\times {\mathrm{C}}_{\mathrm{a}}-9.76\times {\mathrm{C}}_{\mathrm{b}})/209$$

The photosynthetic parameters including net CO_2_ assimilation rate (Pn), stomatal conductance (Gs), and intercellular CO_2_ concentration (Ci) of *yl* mutant and wild-type plants were measured in the greenhouse from 9:00 to 12:00 on a sunny day using the LI-6800 photosynthesis measurement system. Chlorophyll fluorescence was measured using a MINI-PAM. The maximum quantum efficiency of Photosystem II (PSII), Fv / Fm = (Fm–Fo) / Fm (Fo is the minimal and Fm the maximal fluorescence yield in dark-adapted leaves), was measured at the beginning of the day from leaves that were dark-adapted for 30 min in dark.

### Transmission Electron Microscopy (TEM)

The procedure of sample preparation for TEM observation was followed as described by Costa [26]. Leaf samples of wild type and *yl* mutant were prepared for TEM from the 1^st^ expanded true leaf when the second leaf expanded fully. Samples were vacuumized and fixed in 2.5% glutaraldehyde in a phosphate buffer (Solarbio, pH = 7.2) at 4 ◦C for 4 h. The samples were dehydrated in a graded ethanol series, and critical point drying was done using liquid CO_2_ in a critical-point drier Bal Tec CPD 030 and 15 nm gold coated on aluminum stubs in a Sputte Coater Bal-Tec SCD 005. The samples were visualized with a Hitachi TEM system.

### Mapping and identification of the candidate gene of *yl*

A modified MutMap method was adopted for mapping the candidate gene for *yl* [27]. The young leaves of WT and *yl* plants were sampled for DNA extraction, following CTAB method [28]. Equal amounts of DNA with 20 yellow leaf plants and 24 wild type plants from CCMC × *yl* F_2_ population were bulked to generate the mutant type pool and wild type pool. Pair-end sequencing libraries with a read length of 150 bp and insert sizes of approximately 350 bp were subjected to whole-genome re-sequencing with Illumina HiSeq 4000. To obtain consensus sequences, clean reads obtained from two DNA-pools were aligned against the cucumber genome sequence (9930 reference genome Version 3) [29]. The consensus-sequenced reads were used to call SNPs with SAM tools software [30]. Variant calling were performed for all samples by using the Unified Genotyper function in GATK software [31]. SNPs were screened using the Variant Filtration parameter in GATK. InDels were filtered by the Variant Filtration parameter. ANNOVAR was used to annotate InDels and SNPs based on the GFF3 files for the reference genome [32].

Bulked segregation analysis (BSA) was applied for quick identification of the linked markers with *yl* locus. The WT pool and MT pool were generated respectively through mixing equal amounts of DNAs of ten corresponding individuals from the F_2_ population of Hazerd × *yl*. InDels markers were developed based on the re-sequencing results of CCMC and Hazerd, and the SSR markers were detected based on 450 markers distributed on 7 chromosomes [33, 34]. The newly developed markers were firstly filtered between two parental lines and their F_1_ to obtain polymorphic markers. And then the polymorphic markers were used to screen the linked markers with *yl* locus. The framework map for *yl* was constructed with the polymorphic markers in 245 F_2_ plants. A larger F_2_ population containing 737 individuals were applied to further narrow down the candidate region. Besides, one dCAPS marker was designed based on the BSA-seq results and used for genotype assay and allelism test. All primers used in the fine mapping of *yl* are listed in Table S2.

### Cloning and the expression analysis of the CscpFtsY candidate gene

Total RNA was extracted from the root, stem, leaf, male flower (anthesis), ovary (one day before anthesis), cotyledon of the CCMC and *yl* plant by FastPure Plant Total RNA Isolation Kit (Polysaccharides & Polyphenolics – rich) (Vazyme) for cloning the gene and analyzing the relative expression level of *CscpFtsY*. The first strand cDNA was synthesized by HiScript III RT SuperMix for qPCR (+ gDNA wiper) (Vazyme). The cDNA from the leaf of CCMC and *yl* plants was used as a template for cloning the target gene. Primers used for amplification of the full-length cDNA of the *CscpFtsY* gene are listed in Table S2.

qRT-PCR was performed in a 96-well plate using a Bio-Rad CFX 96 Real-Time PCR System with ChamQ SYBR qPCR Master Mix (Vazyme). The cucumber *CsaACTIN2* was used as the reference gene to normalize the gene expression results. The amplification was initiated by heating to 94 °C for 10 min, followed by 40 cycles of 94 °C for 5 s and 57 °C for 30 s. All experiments were performed with three biological replicates and three technical replicates. Gene expression level at different organs was calculated by the basis of the 2^–ΔΔCt^ and the values represented the n-fold difference of *yl* relative to the gene expression of WT. The primers used are listed in Table S2.

### Protein sequence alignment and phylogenetic analysis

Based on the results of gene cloning, the protein sequence alignments were conducted with the DNAMAN software to detect variations between *yl* and WT. Protein sequences of CscpFtsY in 12 species were downloaded from Uniprot database to analyze the phylogenetic relationship. The names of species and accession IDs of protein sequences in NCBI database were as follows: *Cucumis melo var. makuwa* KAA0048987.1, *Cucumis sativus* XP_004133877.1, *Cucurbita maxima* XP_022975096.1, *Vitis vinifera* XP_002269433.1, *Glycine max* XP_040870258.1, *Daucus carota subsp. sativus* XP_017215349.1, *Nicotiana tabacum* XP_016483343.1, *Brassica napus* CDY18901.1, *Arabidopsis thaliana* AAD47910.1, *Escherichia coli K-12* P10121.1, *Oryza sativa Japonica Group* XP_015629030.1, *Zea mays* ACG42442.1. Multiple sequences were aligned by Clustal Muscle, and the neighbor-joining tree was constructed using MEGA X based on a bootstrap test of 1000 replicates.

### Subcellular localization of CscpFtsY

To investigate the distribution of CscpFtsY in cells, the coding sequence of *CscpFtsY* (the stop codon was deleted) was amplified and fused with pGreen vector which contains green fluorescent protein (GFP) to generate *p35s:: CscpFtsY-GFP* vector. The recombinant expression vector was injected into tobacco (*Nicotiana benthamiana*) leaves for subcellular localization, and the pGreen vector was used as a negative control. The infected tobacco plants were cultivated in darkness for 24 h (in darkness) and 48 h (in daylight) to allow the transient expression of *p35s:: GFP* vector and *p35s:: CscpFtsY-GFP* vector. The green fluorescence signals were observed and photographed with the laser scanning confocal microscope.

## Discussion

In this study, we identified a yellow leaf (*yl*) cucumber mutant with reduced Chls and carotenoid contents. According to incomplete statistics, 12 leaf color mutants have been identified in cucumber, and 9 related genes have been cloned [35–46] (Table S1). With the exception of the *ygl1* [39] exhibiting relatively increased carotenoid content, Cucumber mutants with yellow leaves were primarily caused by a deficiency in Chls and carotenoid content. The *yl* mutant not only showed reduced Chl a, Chl b content, and the ratio of Chl a / Chl b, but also expressed increased Chl a / Chl b ratio during leaf growth and development compared with WT. The chloroplast ultrastructure in leaf color mutants were also impaired at different levels which is contrary to earlier report wherethe chloroplast outer membrane of *yyl-1* was intact and non-oval shaped [42]. Compared with the *virescent yellow leaf* (*vyl*) mutant [38], the grana stacks decreased drastically in *yl*. Reduced Chls concentration and compromised photosynthetic machinery in *yl* are featured contributing to diminished photosynthetic capacity in *yl* mutant. Moreover, there were a large number of plastoglobules in the chloroplasts of *yl* while none could be observed in the chloroplasts of CCMC. Plastoglobule plays a role in Chls degradation and phytol recycling, and many thylakoids or chloroplast biogenesis mutants show increased accumulation of plastoglobules [47]. The increased number of plastoglobules may be a sign of increased free-form Chls, which indicated an interruption in the combination of Chls with LHCPs. Leaf color can be easily affected by light intensity and temperature, however, from our observation, *yl* mutant was not sensitive to either.

The application of high-throughput sequencing has made it easier and faster to map candidate genes related to a phenotype [27]. In this study, the initial mapping interval of *yl* traits was obtained by BSA-seq. Based on the confident sequencing results, polymorphic markers were developed according to the initial mapping interval, and *yl* locus was finely mapped by screening recombinants. Finally, through the larger segregation population, the *yl* locus was located close to 100 Kb, and then combined with the BSA-seq file, the SNP in this interval was screened and one SNP was obtained. Then the dCAPS3-1 marker was designed for the SNP, and the genotypes of the recombinants in the interval were identified. It was found that dCAPS3-1 was consistent with their phenotype in recombinants. Then the allele test was carried out by using the dCAPS3-1 marker, and the genotypes of nearly natural 80 cucumber inbred lines (including CCMC and Hazerd) with different ecological types were consistent with those of wild-type parents (Fig. S2). In light of this, we deduced that the SNP (G334A) in CsaV3 3G009150 causes yellow leaves in *yl* mutant. Homology analysis of CsaV3_3G009150 showed that it is a homolog of *cpFtsY* in *Arabidopsis thaliana*, which encodes chloroplast signal recognition particle receptor [24], and these two proteins shared a 84% identity in amino acid sequence.

The chloroplast signal recognition particle (cpSRP) and its receptor, cpFtsY, post-translationally target the nuclear-encoded light-harvesting chlorophyll-binding proteins (LHCPs) to the translocase Alb3 in the thylakoid membrane [48]. The assembly of LHCPs is coordinated with Chls biosynthesis during chloroplast development [22]. Variations in leaf color and anomalies in the photosynthetic system could originate from any obstruction in the transport channel for cpSRP-dependent LHCPs to the thylakoid membrane [23, 24, 49–52]. The structures and functions of cpSRP members have been well-studied in *Arabidopsis thaliana *[53, 54]. The cpSRP receptor, cpFtsY binds peripherally to thylakoid membranes, containing an NG domain that is necessary for GTP binding and hydrolysis [19]. Similar to cpFtsY, cpSRP54 also contains an NG domain that interacts with the NG domain in cpFtsY to promote GTP hydrolysis thus guaranteeing energy supply in the insertion of LHCPs to thylakoid membranes. However, the substitution of G334A changed the hydrophobic amino acid to a hydrophilic amino acid (G112R) in cucumber, and the amino acid multiple sequences alignment analysis of cpFtsY showed that the change located in the conserved N domain of FtsY in *E.coli* (Fig S3). The N domain is packed together by hydrophobic residues that are conserved between members of the SRP family [54, 55]. We speculated that the mutation in N domain changed the function of CscpFtsY and affected the chloroplast development and chlorophyll biosynthesis, thereafter causing the yellow leaf in *yl*.

The cpSRP members demonstrate pleiotropy in their biological roles and are also involved in in the assembly of LHCPs to thylakoid membranes. Since all null Arabidopsis mutants in cpSRP pathway have adequately collected, it is therefore convenient to get a phenotype for comparison. In Arabidopsis, the null single cpSRP (*ffc-cpsrp54* or *chaos-cpsrp43*) mutants are all viable but with some distinct phenotypes. The cpSRP43 null mutant *chaos* showed chlorotic leaves color with an elevated Chl a/b ratio [56], while the cpSRP54 null mutant produced yellow first true leaves that become green 3 to 4 days later, and Chl a/b ratios were unchanged in both yellow and green leaves [57]. In protein levels, there was a selective loss in LHCPs relative to other thylakoid proteins, while many thylakoid proteins were affected in both yellow and green leaves in *ffc*. The double cpSRP43/cpSRP54 mutant showed a more intense defect than any of the single cpSRP43/cpSRP54 null mutant, with pale yellow leaf and a more drastically loss of LHCPs, suggesting cpSRP43 and cpSRP54 contributed to LHCPs integrated in thylakoid membrane in an independent but additive way [50]. The cpFtsY and ALB3 respectively resides in the cpSRP receptor and translocase in the cpSRP pathway [58, 59]. The null cpFtsY mutant showed a similar defect with the double cpSRP43/54 mutant but more moderate defect compared with the null ALB3 mutant, which was lethal on soil [24, 52, 58]. The *cpftsy* mutant, *frd4*, does not induce Fe (III) chelate reductase activity in their roots in response to iron deficiency. Suggesting that cpFtsY may affect Fe (III) chelate reductase activity under iron deficiency [24]. Recent researchers had uncovered that cpSRP43 not only binds LHCPs, but also provides a chaperone domain, substrate-binding domain and chromodomain 2 (CD2), to protect several tetrapyrrole biosynthesis (TBS) proteins from aggregation under heat shock, therefore post-translationally regulates Chls biosynthesis [22]. In this study, the yellow leaf phenotype was identified with a mutation in *CscpFtsY* in cucumber. The TEM observation and subcellular localization result support that CscpFtsY plays a role in chloroplast development. In light of our findings, further research is required to determine the additional role of CscpFtsY in Chls biosynthesis and chloroplast development and that additional investigation is required to identify the flaws in the Chls metabolic and LHCPs targeting pathway.

## Conclusions

The *yl* cucumber mutant displayed intense yellow leaf color at the cotyledon stage and progressively maintained it during its whole developmental stage. The Chls and carotenoid content and photosynthesis capacity were significantly reduced in *yl*. The chloroplasts ultrastructure was severely impaired with less and intermittent grana stacks and more plastoglobules. The isolation and mapping of *CscpFtsY* could help to uncover the role of cpSRP receptor in chloroplast formation and development as well as posttranslational regulation of Chls biosynthesis.

## Supplementary Information

**Additional file 1.**

## Data Availability

The data generated and analysed during the current study are available in NCBI repository under accession PRJNA890006 [https://dataview.ncbi.nlm.nih.gov/object/PRJNA890006]. Other data generated or analyzed during this study are included in the “Supplementary materials” part and available from the corresponding author upon reasonable request. The *yl* material is identified and stored in our laboratory, however, we don’t design a voucher ID number for it. The *yl* material is available from the corresponding author upon reasonable request.

## Abbreviations

BSA: Bulked segregant analysis sequencing
Chls: Chlorophyll a/b
cpSRP: Chloroplast signal recognition particle
cpFtsY: Chloroplast signal recognition particle receptor
EMS: Ethylmethane sulfonate
LHCPs: Light harvesting Chlorophyll a/b proteins
Mutant type: MT
Wild type: WT
