# Supplementary material for: Fine mapping of CscpFtsY, a gene conferring the yellow leaf phenotype in cucumber (Cucumis sativus L.)
Source: BMC Plant Biol. 2022 Dec 6;22:570. doi: 10.1186/s12870-022-03922-0 (PMC9724417; doi:10.1186/s12870-022-03922-0)
Supplement: Supplementary file 1 — Additional file 1. [file 12870_2022_3922_MOESM1_ESM.zip › Supporting information Zha_plain.docx]

**Supporting information**

**Title：Fine Mapping of CscpFtsY, a Gene Conferring the Yellow Leaf Phenotype in Cucumber (*Cucumis sativus* L.)**

Gaohui Zha^1^ · Juan Yin^1^ · Feng Cheng^1^ · Mengfei Song^1^ · Mengru Zhang^1^ · Hesbon Ochieng Obel^1^ · Yi Wang^1^ · Jinfeng Chen^1^ · Qunfeng Lou^*^

^1^ State Key Laboratory of Crop Genetics and Germplasm Enhancement, College of Horticulture, Nanjing Agricultural University, Weigang Street No.1, Nanjing 210095, China

***Corresponding author**: Qunfeng Lou (qflou@njau.edu.cn)

**Author**

Gaohui Zha 2020104064@stu.njau.edu.cn

Juan Yin 2020104065@stu.njau.edu.cn

Feng Cheng [2018204019@njau.edu.cn](mailto:2018204019@njau.edu.cn)

Mengfei Song [2017204016@njau.edu.cn](mailto:2017204016@njau.edu.cn)

Mengru Zhang [2019204035@njau.edu.cn](mailto:2019204035@njau.edu.cn)

Hesbon Ochieng Obel 2019204059@njau.edu.cn

Yi Wang wangyi@stu.njau.edu.cn

Jinfeng Chen [jfc](mailto:jfchen@njau.edu.cn)[hen@njau.edu.cn](mailto:hen@njau.edu.cn)

Qunfeng Lou qflou@njau.edu.cn

**
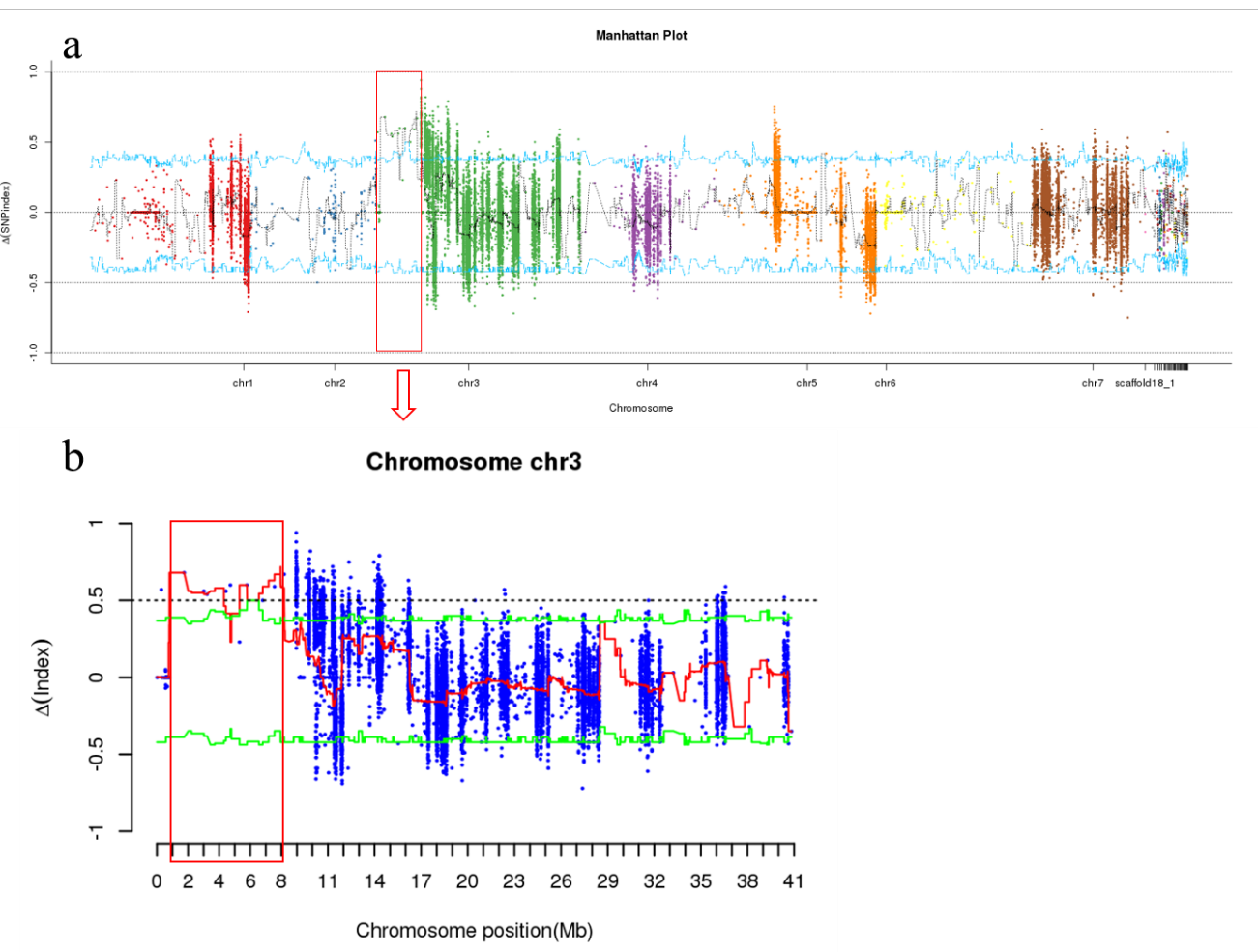
 Fig. S1 The delta SNP manhattan plot of BSA-seq.**

a. ΔSNP index plot (subtracting the SNPindex value of the mutant bulk from the WT bulk) of whole genome, blue line represents the 95% threshold line. b. ΔSNP index plot in chromosome 3, the green line represents the 95% threshold line. Points represent the SNP index, and the two red rectangles indicate the peak exceeding the 95% threshold lines in a and b.

Fig S2. **Linkage analysis and allelism test by dCAPS3-1 based on candidate SNP**

a

b


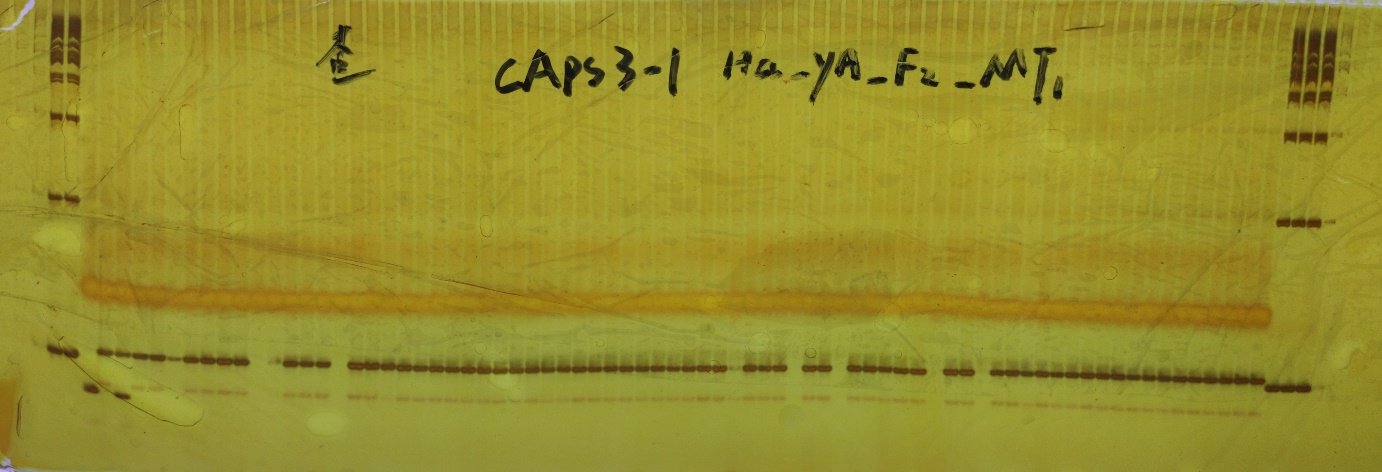

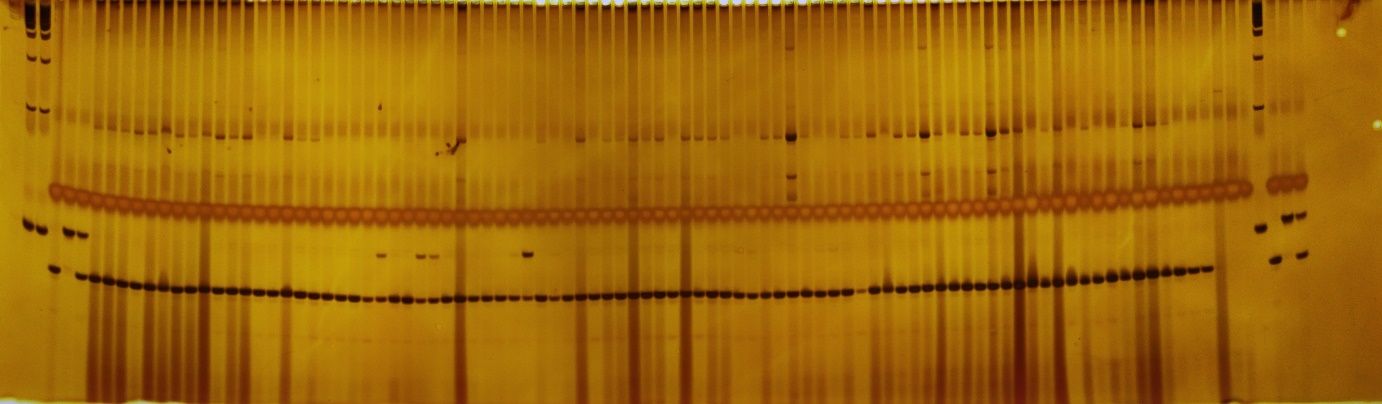


c

WT

*yl*

F_1_

WT

F_1_

*yl*

22

25

26

33

a. Genotype identification by dCAPS3-1 in MT individuals b. Allelism test by dCAPS3-1 in 80 cucumber inbred lines (Should note that 22nd, 25th, 26th, and 33rd these four bonds exhibited F_1_ bond, but their genotypes were the same as WT based on sequencing result, which was shown in c).

**Figure S3. Multiple sequences alignment of cpFtsY**

**
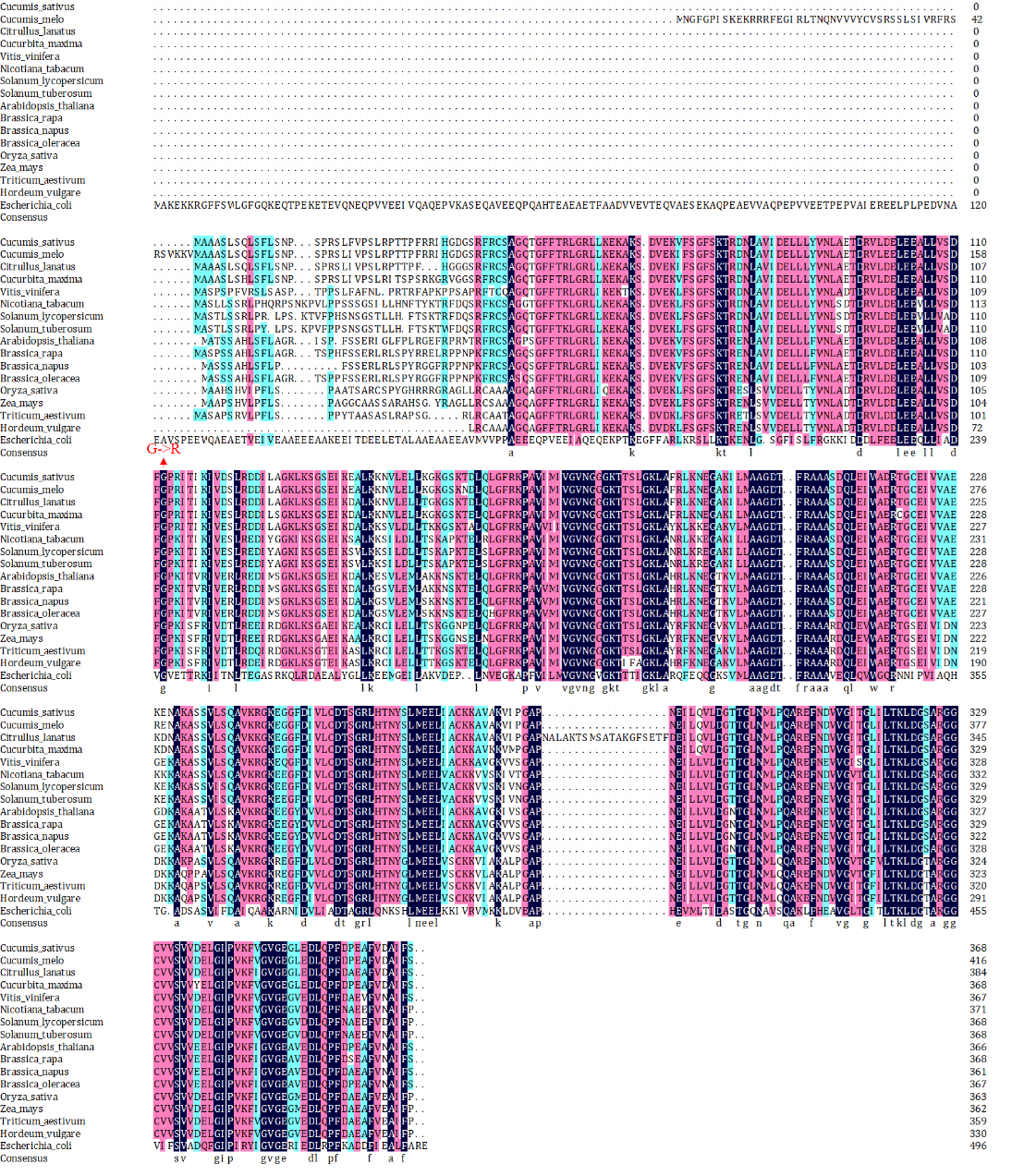
**

**Table S1 The information of cucumber leaf color mutants**

| **Leaf Color** | **Mutation** | **Inherited Pattern** | ***Locus*** | ***Gene ID*** | **Gene Annotation** | **Chr** |
| --- | --- | --- | --- | --- | --- | --- |
| Golden leaf [23] | EMS | Single nuclear recessive | *yp* | *CsChlⅠ* | CHLI subunit of cucumber Mg-chelatase | 6 |
| Virescent leaf [24] | Spontaneous | Single nuclear recessive | *v-1* | *CsaCNGCs* | cyclic-nucleotide-gated ion channel protein | 6 |
| Green-Yellow-White variegation [25] | EMS | Single nuclear recessive | *csvl* | *Csa6G405290* | chorismate synthase | 6 |
| virescent yellow [26] | EMS | Single nuclear recessive | *vyl* | *Csa4G637110* | DnaJ-like zinc finger protein | 4 |
| Yellow-Green [27] | Spontaneous | Single nuclear recessive | *ygl1* | *Csa4M286960*  *Csa4M287550*  *Csa4M288070*  *Csa4M288080* | lipoxygenase isoenzymes | 4 |
| Yellowing [28] | Spontaneous | Single nuclear recessive | *yf* | *Cucsa.099260.1* | cpSRP43 | 7 |
| Yellowing [29] | EMS | Single nuclear recessive | *yl-2* | *Csa6G385090* | Chlorophyllide a oxygenase | 6 |
| Yellow-Young [30] | EMS | Single nuclear recessive | *yyl-1* | *Csa3G836480* | HD domain-containing protein | 3 |
| Virescent [31] |  | Single nuclear recessive | *v-2* | *Csa3G890020* | auxin F-box protein | 3 |
| Yellow [32] | EMS | Single nuclear recessive | *yl2.1* | *Csa2G263900* | plastid isoform of triose phosphate isomerase | 2 |
| Yellow [33] | Tnt1 Transposon | Single nuclear recessive | *yl-5* | *Csa3G588450* | chloroplast 30S ribosomal protein S21 | 3 |
| Albino [34] | EMS | Single nuclear recessive | *al* | *Not Mentioned* |  | 7 |

**Table S2 . All primers’ information used in this study**

| Primer ID | Application | 9930V3.0_Pos | Forward primer 5'-3' | Reverse prime5'-3' | Note |
| --- | --- | --- | --- | --- | --- |
| CsACTIN2 | qPCR |  | ATTGTTCTCAGTGGTGGTTCTAC | CCTTTGAGATCCACATCTGCT | Reference gene |
| qCscpFtsY | qPCR |  | GAAACCGATAGAGTTCTCGACG | ACTACCAGATTTAAGCTTCCCAG | Candidate gene |
| pG-1 | Subcellular localization |  | atctagaggatccccATGGCCGCGGCCTCAC | cggccggtggatcccTGAAAATATGGCATCAACAAAGGC |  |
| Ind3-27 | Mapping | 324948 | CTTTTTCCACACGTTTCTTT | CCCCTAGCTCCTTAATAGAT | InDel |
| Ind3-28 | Mapping | 1277740 | TGTTTCTCCTAACATTTTGC | TGAATTTAGCCTATGCATTC | InDel |
| AInd3-3 | Mapping | 2656159 | TGATATTGAGATGGGTTGTC | TATAAGGGTTGGTTAGGTCT | InDel |
| AInd3-4 | Mapping | 3739510 | GACAATATGAATGAAACCCC | AAATTCACATCTAAGAACGC | InDel |
| AInd3-6 | Mapping | 5524418 | AAACTCATATTGTGGAGTCC | TTCTATTTTGAAGGGTCACT | InDel |
| AInd3-7 | Mapping | 6023890 | GTATTTACAGTTCAGTGTATAAC | TCTTACACCTCTATTTGATACA | InDel |
| yb-Indel3-2 | Mapping | 6968518 | TTTTGTAGTTTCACATTGGC | CTGTACCTGTTGCATCAGAG | InDel |
| AInd3-12 | Mapping | 7067545 | AAAATGGCACCTTGAGATTC | TGTTTGGACCATGAAAATCC | InDel |
| AInd3-14 | Mapping | 7270205 | TTTCGCTGTCACTTTCAAAT | AGATGATCATGGTTCCTAGC | InDel |
| AInd3-15 | Mapping | 7365867 | TGTCCATTACTTGATGCAAG | AGAAAGGAGAAGGTACACTG | InDel |
| AInd3-21 | Mapping | 7426209 | GTGTTTCGTTTGGATCTATT | CCTGCTGATAGAATAAACTG | InDel |
| AInd3-22 | Mapping | 7471383 | ACGAGCATCTATCACTTATAG | CATACACCGTTTACAAAGAT | InDel |
| AInd3-16 | Mapping | 7474497 | TTTCTTGTCAAATCCATTGA | CAAAATATCTCTCGAAAAAGAC | InDel |
| dCAPS3-1 | Allelism test | 7561767 | CTTAATGGTGATCCTTGGCT | ATTTCGGGAGATTGGAGTC | dCAPS,TaqⅠ |
| AInd3-24 | Mapping | 7573226 | TCAGATGATAAACATGCGT | ATGATGACATTTGTTTCGTG | InDel |
| AInd3-18 | Mapping | 7648036 | GATTTTATCGGTCATGTAAATC | CTCAAGGAGAAAACGAGAAT | InDel |
| AInd3-19 | Mapping | 7802738 | TCCTAACTCTCTCAAAGCTT | AGTAATCGACTAGAATGAACT | InDel |
| yb-Indel3-3 | Mapping | 7857562 | TTTTGCGAAGTCGATGTAGG | AGATCATTGAATCTCAGCCG | InDel |
| Ind3-35 | Mapping | 9170070 | CAGAGCAAAAGAATCAGAAG | TTTCCTTAGCTTGCTTCAT | InDel |
| Ind3-36 | Mapping | 10189337 | ACATAAAGTCAGAATAACCAC | TAAATCTAGCAAAAGCGAAG | InDel |
| yb-Indel3-6 | Mapping | 10922249 | TGAACTCTAGGAAACAAAAT | TTCCATAGGACTAACTATTT | InDel |

**Table S3. The annotations of 20 Genes in the fine mapping region**

| Gene ID | Annotation |
| --- | --- |
| CsaV3_3G009010.1 | Remorin family protein |
| CsaV3_3G009020.1 | MYB transcription factor |
| CsaV3_3G009030.1 | ATP-dependent RNA helicase |
| CsaV3_3G009040.1 | Transmembrane protein, putative |
| CsaV3_3G009050.1 | Cleavage and polyadenylation specificity factor subunit 5 |
| CsaV3_3G009060.1 | ABC transporter substrate-binding protein |
| CsaV3_3G009070.1 | LIM domain-containing protein |
| CsaV3_3G009080.1 | Unknown protein |
| CsaV3_3G009090.1 | K(+) efflux antiporter 2, chloroplastic-like |
| CsaV3_3G009100.1 | Bifunctional protein FolD |
| CsaV3_3G009110.1 | Threonine aspartase, putative |
| CsaV3_3G009120.1 | LIM domain-containing protein |
| CsaV3_3G009130.1 | digalactosyldiacylglycerol synthase 2, chloroplastic |
| CsaV3_3G009140.1 | acyl-coenzyme A thioesterase 13 |
| *CsaV3_3G009150.1* | *Signal-recognition particle receptor FtsY* |
| CsaV3_3G009160.1 | BON1-associated protein 2-like |
| CsaV3_3G009170.1 | Unknown protein |
| CsaV3_3G009180.1 | BON1-associated protein 2-like |
| CsaV3_3G009190.1 | BON1-associated protein 2-like |
| CsaV3_3G009200.1 | E3 ubiquitin-protein ligase |

**Table S4 SNPs in the fine mapping region**

| Mutation site |  | Gene | INFO | CCMC | F_2_-MT | F_2_-WT |
| --- | --- | --- | --- | --- | --- | --- |
| upstream;  downstream |  | CsaV3_3G009040;  CsaV3_3G009050 | GT:AD:DP:GQ:PL | 1/1:0,11:11:27:354,27,0 | 1/1:0,22:22:66:880,66,0 | 1/1:0,19:19:57:760,57,0 |
| exonic |  | CsaV3_3G009090 | GT:AD:DP:GQ:PL | 1/1:0,11:11:33:430,33,0 | 1/1:0,17:17:51:670,51,0 | 1/1:0,27:27:72:939,72,0 |
| intronic |  | CsaV3_3G009090 | GT:AD:DP:GQ:PL | 0/1:5,3:8:38:38,0,179 | 0/1:9,5:14:99:158,0,308 | 0/1:14,5:19:75:75,0,440 |
| *exonic* |  | *CsaV3_3G009150* | *GT:AD:DP:GQ:PL* | *0/0:10,0:10:30:0,30,400* | *1/1:0,16:17:45:600,45,0* | *0/1:13,9:22:99:297,0,411* |
| intergenic |  | CsaV3_3G009160(dist=1339),  CsaV3_3G009170(dist=1006) | GT:AD:DP:GQ:PL | 0/1:1,5:6:22:161,0,22 | 0/1:4,17:21:99:580,0,100 | 0/1:3,17:20:31:565,0,31 |
| exonic |  | CsaV3_3G009170 | GT:AD:DP:GQ:PL | 1/1:0,9:9:24:325,24,0 | 1/1:0,26:26:78:1040,78,0 | 1/1:0,25:25:75:1000,75,0 |
| upstream |  | CsaV3_3G009170 | GT:AD:DP:GQ:PL | 1/1:0,14:14:42:539,42,0 | 1/1:0,26:26:69:920,69,0 | 1/1:0,15:15:45:600,45,0 |
| upstream |  | CsaV3_3G009180 | GT:AD:DP:GQ:PL | 1/1:0,7:7:21:270,21,0 | 1/1:0,21:21:63:830,63,0 | 1/1:0,18:18:54:720,54,0 |
| upstream |  | CsaV3_3G009180 | GT:AD:DP:GQ:PL | 1/1:0,9:9:24:320,24,0 | 1/1:0,23:23:63:845,63,0 | 1/1:0,17:17:45:605,45,0 |

Note that 0/0 (example: 0/0:10,0:10:30:0,30,400) means sharing the same genotype as the reference sequence and homozygous, 1/1 means different genotype from reference sequences and homozygous, 0/1 means containing the reference sequence but heterozygous. Only the SNP residing CsaV3_3G009150 was polymorphism between CCMC and F_2_-MT, which can be predicted as the candidate SNP in the fine mapping region.
